# High-throughput identification of interacting protein-protein binding sites

**DOI:** 10.1186/1471-2105-8-223

**Published:** 2007-06-27

**Authors:** Jo-Lan Chung, Wei Wang, Philip E Bourne

**Affiliations:** 1 Department of Chemistry and Biochemistry, University of California, San Diego, Gilman Drive, La Jolla, CA 92093-0743, USA; 2 Department of Pharmacology, University of California, San Diego, Gilman Drive, La Jolla, CA 92093-0743, USA; 3 San Diego Supercomputer Center, University of California, San Diego, Gilman Drive, La Jolla, CA 92093-0743, USA

## Abstract

**Background:**

With the advent of increasing sequence and structural data, a number of methods have been proposed to locate putative protein binding sites from protein surfaces. Therefore, methods that are able to identify whether these binding sites interact are needed.

**Results:**

We have developed a new method using a machine learning approach to detect if protein binding sites, once identified, interact with each other. The method exploits information relating to sequence and structural complementary across protein interfaces and has been tested on a non-redundant data set consisting of 584 homo-dimers and 198 hetero-dimers extracted from the PDB. Results indicate 87.4% of the interacting binding sites and 68.6% non-interacting binding sites were correctly identified. Furthermore, we built a pipeline that links this method to a modified version of our previously developed method that predicts the location of binding sites.

**Conclusion:**

We have demonstrated that this high-throughput pipeline is capable of identifying binding sites for proteins, their interacting binding sites and, ultimately, their binding partners on a large scale.

## Background

Protein-protein interactions are essential to most biological processes, for example, signal transduction, hormone-receptor binding and immunological recognition. These processes comprise complex cellular protein interaction networks that are becoming increasingly accessible in the post-genome era of high-throughput proteomics. Experimental methods such as mass spectrometry, phage display and yeast two hybrid have been developed to quickly identify interactions between proteins in various organisms [1-4]. Concurrently, computational approaches exploiting amino acid properties, genomic and evolutionary information [5-13] have been proposed to determine whether proteins interact or not (binary interactions). While both large scale experimental and computational methods are known to produce many false positive and false negative predictions, the combination of using several methods may provide more reliable results. The idea of using consensus results is not new and has been used in the meta-servers for structure prediction, generating consensus models according to the results of several prediction servers [14]. Here we provide a new approach to the computational prediction of interacting protein-protein binding sites which can contribute to this greater accuracy [1].

Thanks, in large part, to the availability of increasing sequence and structural information, various computational methods have been proposed to identify putative protein-protein binding sites utilizing evolutionary relationships [15-19], properties of surface patches [20,21], residue hydrophobicity [22], etc. Recently, machine learning approaches such as neural networks [23-26], support vector machines [27-31] and Bayesian network [32] have been used to distinguish interface residues from non-interface residues based on sequence and structural properties.

All of these methods locate binding sites from protein surfaces, but none of them provide information about their binding partners (binding specificity). Therefore, methods that identify interacting protein binding sites are necessary. Inherently these methods would then allow more reliable determination of binary interactions. Docking approaches provide this information by predicting the binary complex of two known structures based on energetic or geometric complementary [33,34]. However, long computation time is often required to determine each putative complex and most docking approaches are limited to rigid protein model analysis. Homology modeling [35] and multimeric threading [36] build an atomic model of a complex based on a template structure using sequence alignments. These two methods have been tested on large scale data sets [37,38]. They both rely on the limited number of structure templates of complexes [39] and usually require sequence identity above 30% between homologs [40,41]. Aytuna et al. [42] predicted protein-protein complexes by seeking pairs of proteins that share structurally and evolutionarily conserved residue similarity to 67 template interfaces. Pazos et al. [13] utilized correlated mutation for determining pairs of proteins that are likely to bind and also identified binding sites concurrently. Although structural information is not required for this method, a large set of multiple sequence alignment for each possible pair of proteins is needed.

**Figure 1:**
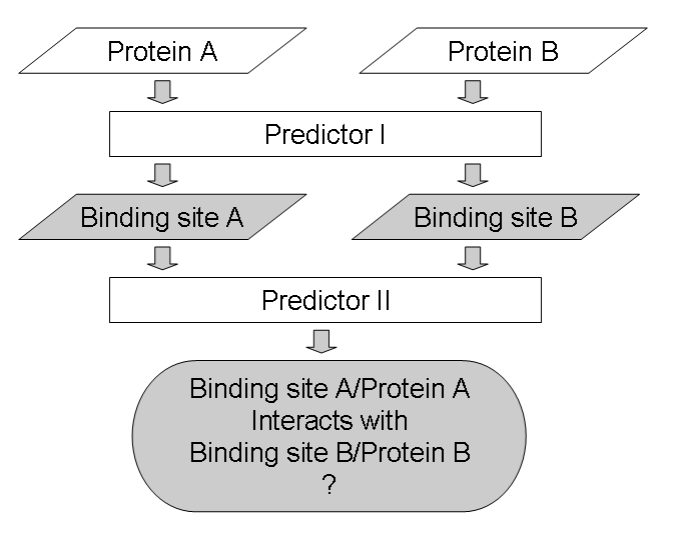
The pipeline for the identification of protein binding sites and their binding partners. Predictor 1 can be any method that predicts the location of protein binding sites. Predictor 2 is the method presented in this study that identifies the interacting binding sites.

In this study, we introduced a new method to determine whether two binding sites interact by using machine learning techniques. A support vector machine was trained on a data set designed to capture the underlying principles of complementary information across protein interfaces. By testing on a non-redundant data set composed of 584 homo-dimer and 196 hetero-dimer structures taken from the Protein Data Bank (PDB [43]), we showed that our method successfully identifies interacting binding sites on a large scale without the constraint of using structure templates. We subsequently built a high-throughput pipeline combining this method with a modified version of our previously developed method that identifies the location of binding sites. As shown in Figure 1, the putative binding sites of two proteins are first located from the protein surfaces and then it is determined if they interact with each other. With this pipeline, we are able to identify both binding sites and binding partners simultaneously.

## Results and discussion

### The contact preferences of interface residues

**Figure 2:**
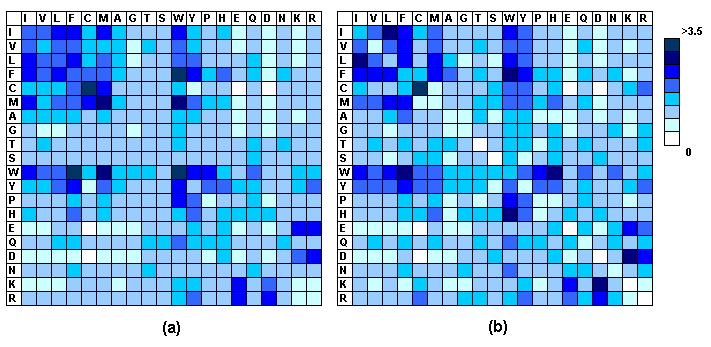
The amino acid contact preferences for (a) homo-dimer and (b) hetero-dimer interfaces. The amino acids are listed according to hydrophobicity [64]. The preferences were calculated with respect to the distribution of interface residues.

A data set consisting of 584 homo-dimers and 196 hetero-dimers with sequence identity below 30% was compiled from the PDB [43]. To understand the contact preferences of interface residues, statistical analysis was performed on the 105871 contacting interface residue pairs derived from the data set. Figure 2 plots the preferences of these contacts formed in homo-dimers and hetero-dimers with respect to the distributions of interface residues. The contact preference was the ratio of the observed contact frequency over the expected contact frequency (see methods for details). Some commonality was observed for these two types of interfaces. As expect, and consistent with previous studies [44,45], the contacts between positively and negatively charged residues were favored and those between residues with the same charge were underrepresented. In addition, there was a relatively high tendency for the interactions between hydrophobic residues. Bogan et al. [46] has reported that, using alanine-scanning mutational analysis of protein interfaces, aromatic residues tryptophan and tyrosine are the two most common amino acids in interface hot spots. In our data set, aromatic residues (tryptophan, tyrosine and phenylalanine) were also found to participate in highly preferred contacts. Some preferred associations reported by previous studies, such as contacts between tryptophan and proline, between phenylalanine and isoleucine, were also observed [47,48].

Ofran et al. have reported significant differences in residue composition and contact preferences between interfaces of hetero-obligomers, hetero-complexes, homo-obligomes, and homo-complexes [49]. In our data set, some differences between homo-dimer and hetero-dimer interfaces have also been observed. There was a higher tendency for hydrophobic-hydrophobic interactions in homo-dimers. On the other hand, more salt bridges and fewer contacts between residues with the same charge were preferred in hetero-dimers.

**Figure 3:**
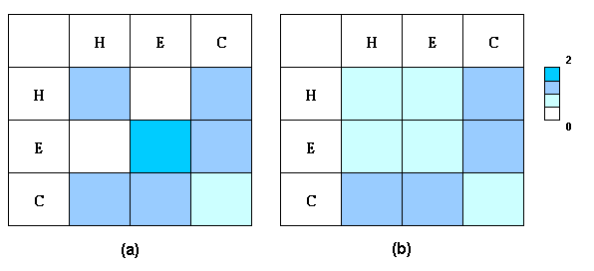
The residue contact preferences in terms of secondary structure properties for (a) homo-dimer and (b) hetero-dimer interfaces. H: alpha helix, S: beta strand, C: others, including coil regions. The preferences were calculated with respect to the distribution of interface residues.

**Figure 4:**
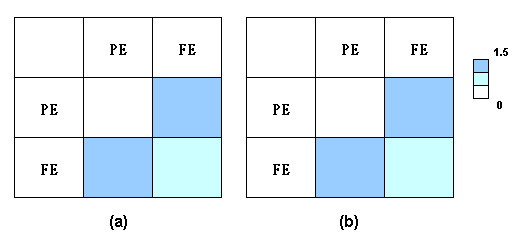
The amino acid contact preferences in terms of the extent of water exposure for (a) homo-dimer and (b) hetero-dimer interfaces. PE: partially exposed (40% > ASA >= 15% of a residue's nominal maximum area), FE: fully exposed (ASA >= 40% of a residue's nominal maximum area). The preferences were calculated with respect to the distribution of interface residues.

Figure 3 plots the distribution of interactions between interface residues with different secondary structure properties (alpha helix, beta strand, and others, including coil regions). Alpha helix-alpha helix and beta stand-beta strand contacts were more preferred in homo-dimers than in hetero-dimers. These contacts are supposed to provide tight packing across protein interfaces. Figure 4 illustrates the interactions between interface residues with different levels of exposure to water (fully exposed and partially exposed). A high preference was found for contacts between fully exposed and partially exposed interface residues. No significant differences were observed for contact preferences between residues with a different extent of water exposure between homo-dimer and hetero-dimer interfaces.

Several previous studies have provided detailed analysis of interaction preferences of different types of protein-protein interfaces in terms of amino acid, secondary structure or other properties [47-51]. The results of those previous studies and our studies show some variations because of the different composition of data sets and the definition of the interface residues. Nevertheless, the survey presented here indicates that information from sequence profile, secondary structure and accessible surface area (ASA) may be useful discriminators for defining contacting interface residues and can be captured by SVM predictors.

### Identification of interacting binding sites using support vector machines

**Figure 5:**
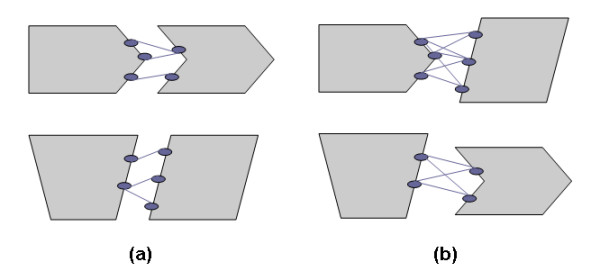
Training Classes of the SVM predictor (a) Positive training class: residue pairs across two interacting binding sites with a distance < 5Å between any of their respective heavy atoms (b) Negative training class: any possible residue pairs between two non-interacting binding sites.

**Figure 6:**
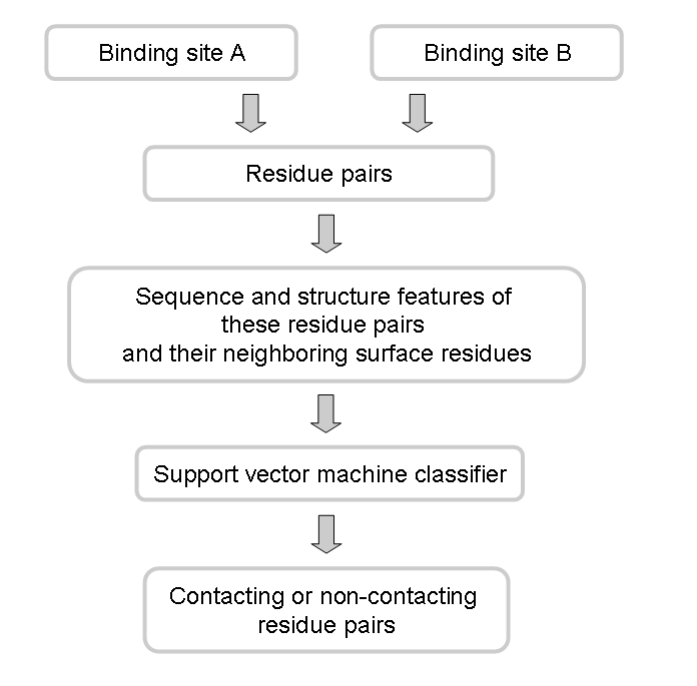
**The prediction of contacting residue pairs**. Proteins A and B are predicted to interact with each other if the percentage of the predicted contacting residue pairs reaches a certain threshold (see methods for details).

It has been reported that proteins and their interaction partners have undergone compensating mutations to maintain interaction specificity [52]. Changes of sequence signatures in one partner's binding surface are complemented by an appropriate change in sequence signatures of its interaction partner [53]. Structural complementarity between associating interfaces has also been observed by previous researchers [54]. In this study, a support vector machine (SVM) was trained to predict whether two binding site interact with each other using the sequence and structural information extracted from the dimer interfaces. Thus the SVM should capture signal associated with compensatory mutations. The machine was trained with different combinations of sequence profile, secondary structure and ASA for residues in contact across interacting binding sites (positive class) and non-contacting residues across non-interacting binding sites (negative class) (Figure 5). The input features of the spatially neighboring residues of the interacting and non-interacting residues were also entered into the SVM. Once training had completed the machine was ready to be used for actual testing. The testing methodology worked as follows (Figure 6): two binding sites were predicted to interact if the number of predicted contacting residue pairs reached a certain threshold. This threshold value was derived from the percentage of the total possible residue pairs between them (see methods for details).

### Prediction performances

**Figure 7:**
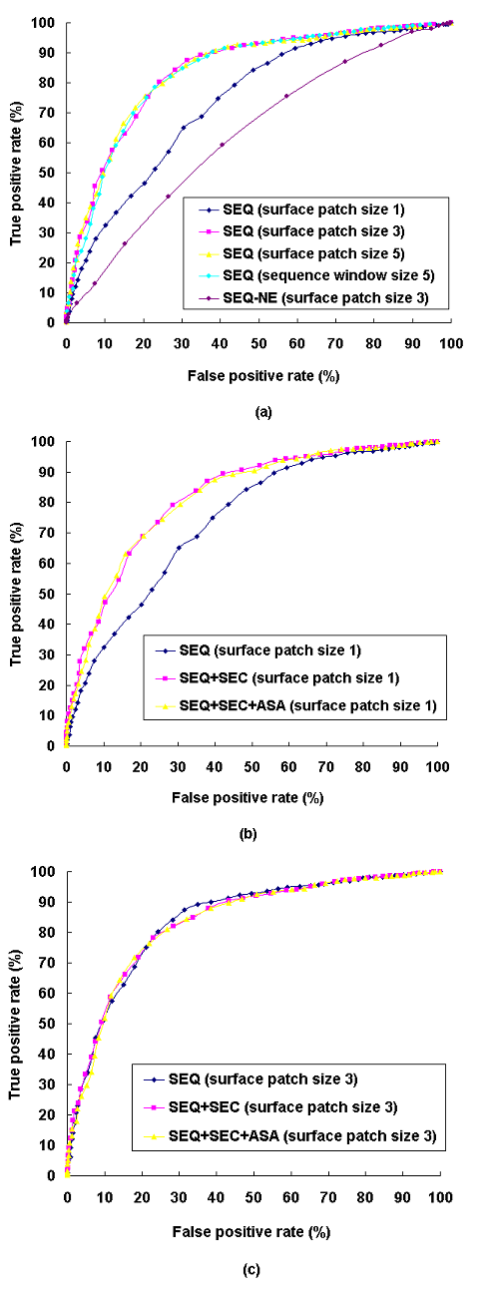
ROC curves for the prediction of interacting and non-interacting protein binding sites using different input features. (a) Predictions using only sequence profile/sequence information but different surface patch or sequence window sizes. Predictions using different combinations of sequence profile, secondary structure, and ASA when (b) surface patch size is 1 (c) surface patch size is 3. SEQ: sequence profile; SEQ-NE: sequence information only (without the evolutionary information provided by homologous sequences); SEC: secondary structure; ASA: accessible surface area.

We first performed 2-fold cross validation on known interacting binding sites derived from crystal structure complexes in our data set using sequence profile data as the only input feature. The data set was randomly separated into two subgroups with an equal number of dimers. Two training and testing processes were performed. For each run, while one subgroup was used as the training set, the other subgroup was used as the test set. As presented by areas under the ROC curves in Figure 7(a), by increasing the surface patch size from 1 to 3 (that is, including 2 spatially nearest surface residues, see methods for details), the prediction performance improved significantly. However, by further expanding the patch size to 5 (that is, including 4 spatially nearest surface residues), no additional improvement was observed. Furthermore, to investigate if the evolutionary information provided by homologous sequences is necessary, we carried out a trial input with only sequence information instead of sequence profile information. As shown in Figure 7(a), without using this evolutionary information, the prediction performance deteriorated significantly.

We then evaluated the prediction method exploiting structural information. At a surface patch size of 1 (Figure 7(b)), incorporating information of secondary structure with the sequence profile increased the prediction accuracy significantly. However, further incorporation of information on the accessible surface area did not result in any additional improvement. As we increased the patch size to 3 (Figure 7(c)), we noticed that secondary structure and ASA had no impact on prediction. Therefore, for this study, we chose a sequence profile with a patch size 3 as the default input features.

**Figure 8:**
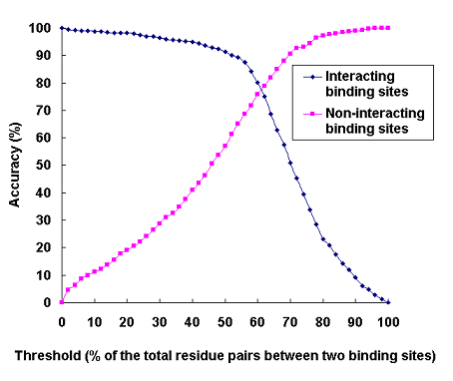
Prediction accuracy for interacting and non-interacting protein binding sites. Two protein binding sites were predicted to interact with each other if the percentage of the predicted contacting residue pairs reached a certain threshold.

Figure 8 plots the prediction accuracy versus the threshold when the default input features were used. The data samples were taken for every 2% increase in threshold. As expected, less interacting binding sites and more non-interacting binding sites were identified as the threshold increased. The average accuracy for interacting and non-interacting binding sites reached the highest value when the threshold was set to 56% of the total possible residue pairs. With this threshold, 87.4% of interacting binding sites and 68.6% of non-interacting binding sites were correctly assigned (Table 1).

**Table 1:** The prediction performances

	Accuracy for interacting binding sites, also known as recall (%)	Accuracy for non-interacting binding sites (%)	Average accuracy (%)	Precision (%)
Mix^1^	87.4	68.6	78.0	73.6
Mix^1 ^(sequence window)	88.7	63.3	76.0	70.8
Mix^1 ^(putative binding sites)	87.3	67.6	77.4	72.9
Homo-dimers^2^	96.4	67.5	81.9	74.8
Hetero-dimers^2^	66.3	62.8	64.5	64.0

^1 ^The mixed data set of homo-dimer and hetero-dimer interfaces.

^2 ^The data set of homo-dimer and hetero-dimer interfaces were separately trained and tested.

The prediction was performed using the default input features (sequence profile with a surface patch size 3).

The surface patch used here included only the two nearest surface residues, which are very likely to be located in the same sequence segment. For this reason we further performed a trial using the sequence profile with a window size 5 in sequence (that is, including 4 sequentially nearest residues). As expected, the ROC curves in Figure 7(a) indicates that the predictor using sequence information only was able to perform similarly to the predictor using a patch size of 3. At a threshold of 56%, 88.7% of interacting binding sites and 63.3% of non-interacting binding sites were correctly assigned. When the average accuracy reached its maximum (threshold: 64%), 78.5% of interacting binding sites and 76.9% of non-interacting binding sites were correctly assigned.

**Figure 9:**
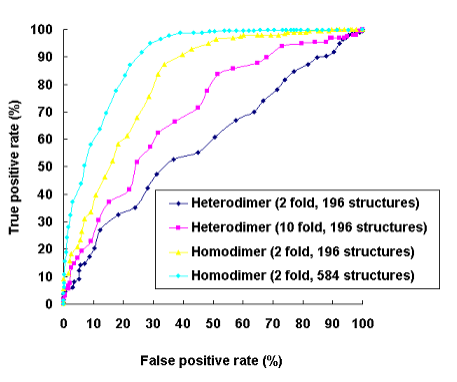
Comparison of the ROC curves for the prediction of interacting and non-interacting protein binding sites between homo-dimer and hetero-dimer interfaces using training data of different sizes.

The data set comprised 584 homo-dimers and 196 hetero-dimers. Using our default predictor, when the accuracy reached its maximum (at threshold 56%), up to 96.1% of the homo-dimers were correctly predicted while only 61.7% of the hetero-dimers were identified. Is this phenomenon attributed to the difference of residue contact preferences between homo-dimer and hetero-dimer interfaces? To answer this question, we trained and tested these two types of interfaces separately. The results showed that homo-dimers were more accurately predicted than hetero-dimers (Figure 9). To evaluate whether this was caused by the larger amount on data available for homo-dimers, 196 homo-dimers were randomly selected and 2 fold cross validation was performed on this subset. Even based on equal data size, homo-dimer interfaces were still significantly better predicted than hetero-dimer interfaces. When we compared the prediction accuracy of homo-dimers of various data set sizes, we found that the larger data set with 584 homo-dimers outperformed the smaller data set of 196 homo-dimers (Figure 9). On the other hand, to improve the prediction for hetero-dimer by using larger training data, we performed 10 fold cross validation on hetero-dimers. The data set was randomly divided into 10 subgroups. For each run, 9 subgroups were combined as the training set and the other subgroup was used as the test set. The resulting prediction was better than using 2 fold cross validation (Figure 9). In summary, our method correctly predicted 96.4% of interacting binding sites and 67.5% of non-interacting binding sites for homo-dimers and 66.3% of interacting binding sites and 62.8% of non-interacting binding sites for hetero-dimers (Table 1). In the future, better predictions, especially for hetero-dimers, is expected as more training data become available.

In this study, two binding sites were predicted to interact with each other if >56% of the total possible residue pairs between them were predicted to be in contact with each other. Raising the threshold increases the precision but decreases the recall (assigns less interacting binding sites and more non-interacting sites) and vice versa. In the data set, the contacting residue pairs for two interacting binding sites constituted, on average, only approximately 8% of the total possible residue pairs. Therefore, the threshold (56%) selected above seem to be very high. However, when we considered the 3 spatially nearest residues of each any two contacting residues to be in contact with each other across the interface, the fraction of contacting residue pairs out of the total possible residue pairs increased to 47%.

**Figure 10:**
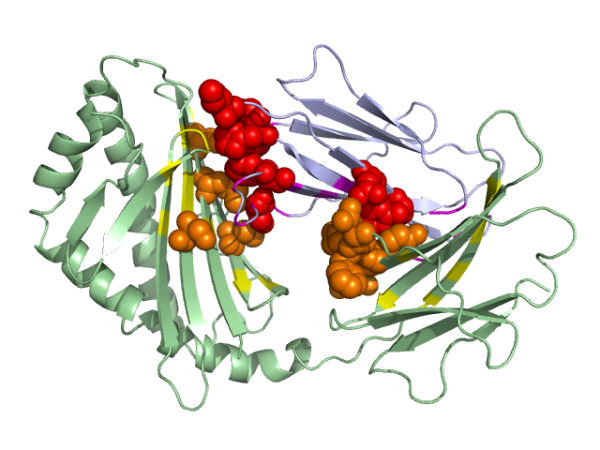
The important contacting residues across the heavy chain and the light chain of the CD1d1 complex (PDB code: 1CD1) assigned by our predictor. The high scoring residues at the binding site (yellow) of the heavy chain (green) were colored orange and presented as spheres. The high scoring residues at the binding site (purple) of the light chain (light blue) were colored red and presented as spheres.

**Figure 11:**
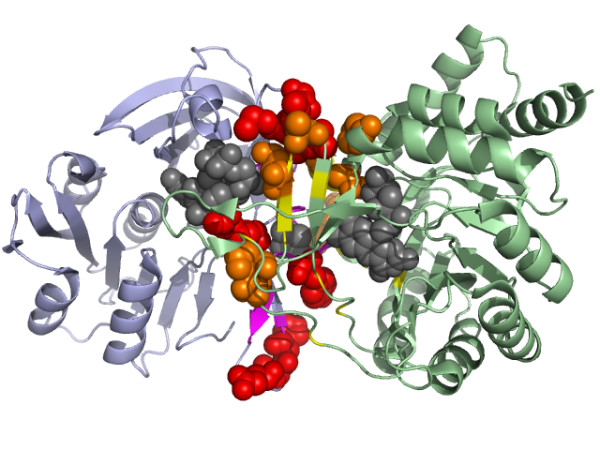
The important contacting residues across the PyrDB and the PyrK subunits of dihydroorotate dehydrogenase B (PDB code: 1EP1) assigned by our predictor. The high scoring residues at the binding site (yellow) of the PyrDB subunit (green) were colored orange and presented as spheres. The high scoring residues at the binding site (purple) of the PyrK subunit (light blue) were colored red and presented as spheres. Three cofactors, FMN, FAD and the [2Fe-2S] cluster were colored gray and presented as spheres.

**Figure 12:**
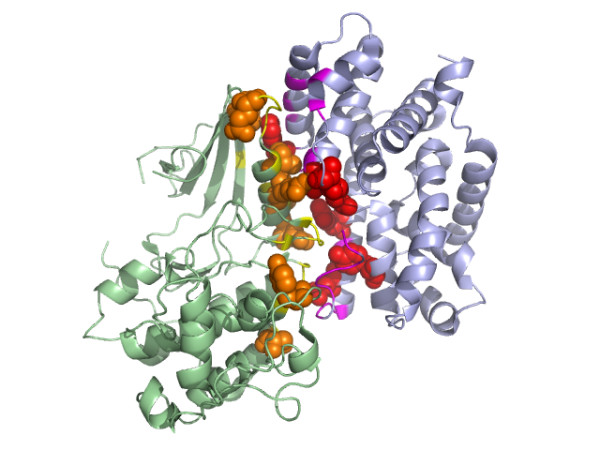
The important contacting residues across protein kinase cdk2 and cyclin (PDB code: 1F5Q) assigned by our predictor. The high scoring residues at the binding site (yellow) of cdk2 (green) were colored orange and presented as spheres. The high scoring residues at the binding site (purple) of cyclin (light blue) were colored red and presented as spheres.

Figure 10 is an example of the prediction of interacting binding sites, showing the interaction between the heavy chain and light chain of the CD1d1 complex. CD1 is a family of non-polymorphic cell surface glycoproteins which fold very much like MHC class I molecules [55]. The high scoring residues were mapped onto the structure. The high scoring residues were those involved in at least 3 predicted contacts, each of which had a SVM predicted score > 0.9. We found that most of these high scoring residues were clustered across the interfaces. Figure 11 illustrates the interface between PyrDB and the PyrK subunit of the flavor protein dihydroorotate dehydrogenase B. Three cofactors (FMN, FAD and [2Fe-2S] cluster) responsible for the transfer of electrons are located in the middle of the interface [56]. The high scoring residues assigned by our predictor were all located on the edge of the interface and the majority of them were close to each other. Figure 12 shows the prediction on the interaction between protein kinase cdk2 and cyclin. Most of the high scoring residues of cdk2 were located near the PSTAIRE helix, which was reported to be an important binding region for cyclin [57].

### Predictions on putative binding sites

A pipeline was built to test if two putative binding sites would interact with each other (Figure 1). Given two proteins A and B, the pipeline first identifies the putative binding site of each protein (with predictor I) and then identifies the interaction between the two putative binding sites (with predictor II, which is the method presented in this study). This pipeline is able to provide information on both the location of binding sites and their binding partners.

Putative binding sites of individual components of each complex in our data set were determined by a method modified from our previous work, using sequence and structural information [29] (predictor I, see methods). With this method, the recall was 65.6% and the precision was 45.2% at the residue level. The results are summarized as follows: 49.81% of the binding sites were precisely predicted, 71.30% of the binding sites were correctly predicted and 23.6% of the binding sites were partially covered by the predicted residues. If at least 70% of the residues at a site were identified, we defined this to be precisely predicted. If at least 50% of the residues at a site were identified, we considered this to be correctly predicted.

**Figure 13:**
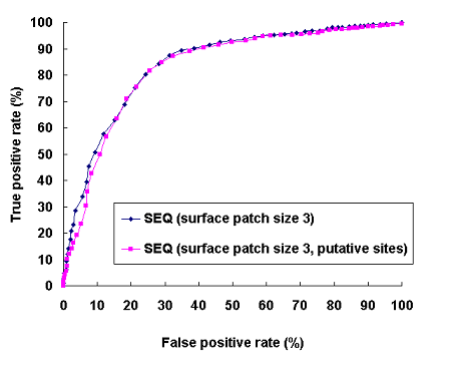
Comparison of the prediction performances based on known binding sites and putative binding sites using sequence profile with a surface patch size of 3 as input.

The interactions of these putative binding sites were further tested using our method (predictor II in Figure 1). Although we used putative binding sites instead of known binding sites, the prediction performance only dropped slightly (Figure 13). At a threshold of 56%, the predictor identified 87.3% of interacting binding sites and 67.6% non-interacting binding sites.

**Figure 14:**
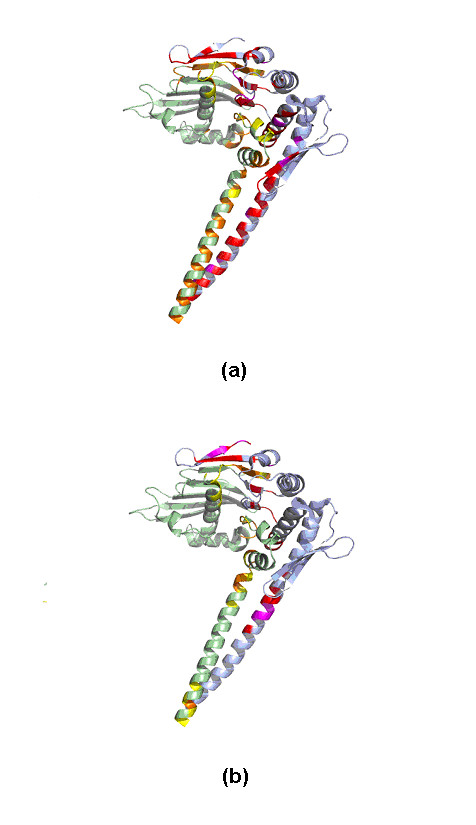
The important contacting residues across the N-terminal and the C-terminal segments of Rad50 abc-ATPase (PDB code: 1II8) assigned by our predictor based on (a) known binding sites and (b) putative binding sites. The high scoring residues at the binding site (yellow) of N-terminal segment (green) were colored orange. The high scoring residues at the binding site (purple) of C-terminal segment (light blue) were colored red.

It is expected that if the putative binding sites are accurately predicted and hence identical to the known binding sites, the resulting prediction of the interaction will be the accurate. However, in most cases, the binding sites are not completely identified, so why were the predicted interactions so good? The following examples might help to explain why the results based on putative binding sites were very close to those based on known binding sites. For Rad50 abc-ATPase [58], most of the interface residues between the N-terminal and C-terminal segments involve high scoring contacts predicted by predictor II (Figure 14(a)). Figure 14(b) illustrated the prediction based on putative binding sites. Although the interface between the N-terminal and C-terminal segments was only partially identified by predictor I, there were still enough high scoring contacting residues at these putative sites identified by predictor II that allowed us to determine the association between these two segments.

**Figure 15:**
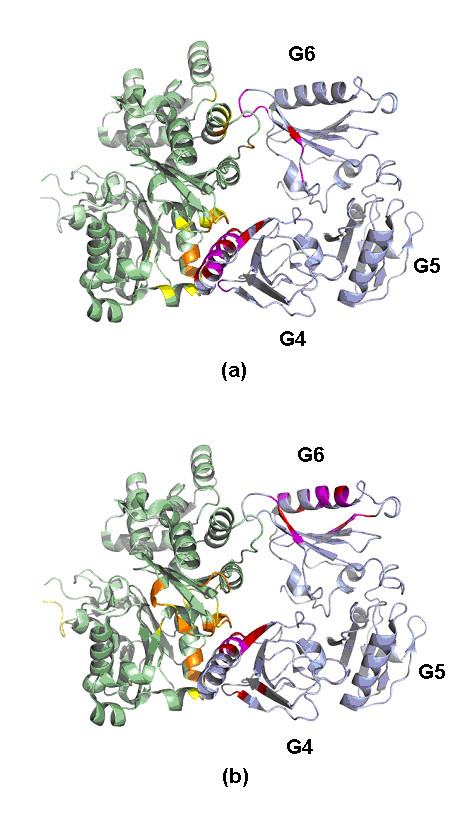
The important contacting residues across gelsolin G4–G6 domains and actin (PDB code: 1H1V) assigned by our predictor based on (a) known binding sites and (b) putative binding sites. The high scoring residues at the binding site (yellow) of actin (green) were colored orange. The high scoring residues at the binding site (purple) of gelsolin G4–G6 domains (light blue) were colored red.

The interaction between actin and gelsolin G4–G6 domains [58] is presented in Figure 15. In Figure 15(b), Predictor I only identified the interface between the G4 domain and actin. However, the high scoring residues identified by predictor II across this interface were sufficient to determine the binding between these two molecules. The falsely predicted binding site in the G6 domain assigned by predictor I caused predictor II to make some erroneous predictions of high scoring contacting residues in this region. This might arise since the fact that both G4 and G6 domains belong to the gelsolin repeat family, so that the false positives on the helix of the G6 domain were predicted to contact actin via the site interacting with the G4 domain.

The pipeline consists of binding site identification (predictor I) and subsequent prediction of whether two of these identified sites interact (predictor II). Since predictor II depends little on spatially neighboring residues and is mostly sequence dependant, a sequence only predictor I could be substituted [19,42].

## Conclusion

We have developed a new method to predict interacting protein binding sites using a machine learning technique. To the best of our knowledge, this method is the first trial that predicts interacting binding sites without the constraint of using structure templates. An SVM was trained to learn the complementary information across interfaces and has been tested on a data set consisting of 584 non-redundant homo-dimer and 198 hetero-dimer interfaces. Our predictor successfully identified 87.4% of the interacting binding sites and 68.6% of the non-interacting binding sites. Separate training and testing on homo-dimers and hetero-dimers showed different prediction results, which might be caused by the differences in residue contact preferences between these two types of interfaces. For homo-dimers, 96.4% of the interacting binding sites and 67.5% of the non-interacting binding sites were correctly identified. For hetero-dimers, 66.3% of the interacting binding sites and 62.8% of the non-interacting binding sites were correctly identified. Better predictions are expected as more structures are determined and the number of homo-dimer and hetero-dimer complexes upon which to train increases.

We built a pipeline combining the method discussed here to a modified version of our previously developed method that identifies the location of binding sites. Taking both predictors together we showed that the prediction accuracy that were based on putative binding sites only decreased slightly over accurately known sites. Thus the pipeline enables the simultaneous prediction of binding sites and binding partners, identifying 87.3% of the interacting binding sites and 67.6% of the non-interacting binding sites in our data set. In the future, the pipeline can be used to search new protein binding sites and interactions in various biological systems, and therefore build interaction networks based on interaction details between proteins. It can also be used to validate existing networks.

At this time it is difficult to compare the results presented here with those of other methods since each uses different training sets and there is a lack of a common test set [41]. In addition, most existing methods have not been tested on negative data and hence prediction statistics (precision, recall, etc.) were not provided. Nevertheless, different methods have different limitations and exploit different information to various extents. For example, most docking procedures are computational expensive, homology modeling and multimeric threading rely on the availability of complex structure templates and correlated mutation methods need a large set of sequence alignment for each possible protein pair. Current efforts are directed at attaining higher prediction accuracy through incorporation of additional information such as local interface geometry or water mediated interactions into our predictor.

## Methods

### Data set

A non-redundant data set of dimer complexes was compiled using the method of Zhou et al. [26] modified as follows. All non-NMR multiple-chain protein entries with resolution better than 3.5 Å were collected from the PDB (March, 2004) [43]. For each entry, two chains were selected as an interacting protein pair if both have more than 20 residues that formed interfacial contacts with each other. A residue was considered to form an interfacial contact if the distance between any of its heavy atoms and any heavy atoms of its interacting proteins were <5 Å. The pairs containing chains with < 80 amino acids or SCOP class >= 8 were then filtered out.

Each of the collected chains was further compared against all other chains by BLAST. Chains were assigned to the same cluster if the sequence identity was > 30% and > 90% of the amino acids were aligned. All interacting protein pairs were mapped to these clusters and the representative pairs were selected. In order to consider dimers only, the representative pairs with chains interacting with more than one chain were discarded. Homo-dimers with both chains having > 30 interface residues and hetero-dimers with both chains having > 20 interface residues were collected, in order to roughly exclude those from crystallographic complexes [30]. This resulted in a non-redundant data set of 584 homo-dimers and 196 hetero-dimers. The data are available upon request from the authors.

### The calculations of contact preferences of interface residues

We have surveyed the preferences of contacts between different groups of interface residues. We classified the residues into 20 groups in terms of amino acids, 3 groups in terms of secondary structures (alpha helix, beta strand, and others, including coil) and 2 groups in terms of the extent of water exposure (fully exposed: ASA >= 40% of a residue's nominal maximum area [59]; partially exposed: 15% <= ASA < 40%). The secondary structure and ASA of residues for each protein chain were calculated using the DSSP program [60] with the coordinates of a single chain obtained from the corresponding complex structure.

The contact preference (*L*) for interface residues from group a and group b was calculated as follows [49]:

*L*(*a*, *b*) = *F*_*observed*_(*a*, *b*)/*F*_*expected*_(*a*, *b*)

where the observed contact frequency was defined as:

*F*_*observed*_(*a*, *b*) = *N*_*observed*_(*a*, *b*)/*N*_*total*_

and *N*_*observed*_(*a*, *b*) was the number of contact residue pairs between residue group a and group b. *N*_*total *_was the total number of all contacting residue pairs. The expected contact frequency was defined as:

*F*_*expected*_(*a*, *b*) = *F*(*a*) × *F*(*b*)

where *F*(*a*) and *F*(*b*) were the frequency of residue group a and group b at interfaces respectively. In this study, a residue was defined as a surface residue if its ASA was at least 15% of its nominal maximum area [59]. A surface residue was defined to be an interface residue (residue at a binding site) if it formed an interfacial contact. The definition of interfacial contact was described in the data set section.

### Support vector machine (SVM) classifiers

The SVMs were trained to predict if two binding sites interact with each other. The SVM software used in this study was SVM^*light *^[61]. The radial basis function exp(-*γ*||*b *- *a*||^2^) was chosen as a kernel with *γ *= 0.01 and regularization parameter C = 10.

**During the training process **(Figure 5), two interface residues, each from interacting binding sites, were considered to form a contacting residue pair (positive class) if the distance between any of their respective heavy atoms was less than 5 Å. A non-contacting residue pair (negative class) was defined as any possible interface residue pair between two non-interacting protein binding sites (binding sites from two non-interacting protein chains). Non-interacting protein chains were generated from our data set having determined that two proteins were not reported to be in the same cellular location as defined by the UniProt database [62]. Since the number of non-interacting protein pairs greatly outnumbered the number of interacting protein pairs, we randomly selected a small portion out of the large pool to be representative data, making the number of non-interacting protein pairs equal to the number of interacting protein pairs. For example, for the combined data set of homo-dimers and hetero-dimers, there were 780 interacting protein pairs. The number of all possible non-interacting protein pairs was 460070. 780 non-interacting protein pairs were further randomly chosen from this pool.

To reduce the data redundancy and training time, for each binding site residue with multiple contacting residues at the other site, only the pairing with the smallest distance was selected to be included in the positive training set. Since there were many more non-interacting residue pairs than interacting residue pairs, a set of non-interacting residue pairs was randomly selected so that the ratio of positive to negative data was 1:1.

The SVM was fed two surface patches, each included a residue of an interface residue pair and its n spatially nearest surface residues (n was an adjustable parameter). The input features were different combinations of sequence profile, secondary structure and accessible surface area of residues in these 2 surface patches. If all three input features were used and surface patch size was set to 3, each residue pair was encoded as a feature vector with a dimension of 2 × 3 × 24: 2 × (the surface residue to be predicted + 2 nearest neighbors) × (20 amino acids + accessible surface area + 3 types of secondary structure). The sequence profiles were obtained from 3 iterations of a PSI-BLAST search against the NCBI non-redundant database (NR) with e = 0.001 and h = 0.001 [63]. The 3 categories of secondary structure were: alpha helix, beta sheet, and others, including coil regions (encoded 1 if it was in this category and -1 if it was not). All input values were scaled between -1 and 1.

**During the testing process **(Figure 6), two binding sites A and B were predicted to interact with each other if the number of positively predicted residue pairs between them were above a certain threshold:

*N*_*predicted *_≥ *P*% × *N*_*total*_

where *N*_*predicted *_is the number of positively predicted residue pairs. *N*_*total *_is the number of total possible residue pairs between binding sites A and B. Given the threshold, the prediction performance was measured as follows:

*Precision *= *TP*/(*TP *+*FP*)

*Accuracy for positive class, or recall, or true positive rate *= *TP*/(*TP *+ *FN*)

*Accuracy for negative class *= *TN*/(*TN *+ *FP*)

*False positive rate *= *FP*/(*FP *+ *TN*)

*Average accuracy *= (*TP *+ *TN*)/(*TP *+ *TN *+ *FP *+ *FN*)

where *TP *is the number of correctly predicted interacting binding sites, *TN *is the number of correctly predicted non-interacting binding sites, *FP *is the number of non-interacting binding sites incorrectly predicted to be interacting and *FN *is the number of interacting binding sites incorrectly predicted to be non-interacting.

### Identification of the putative binding sites

The putative protein-protein binding sites were determined by a method modified from our previous work [29]. A SVM was trained to locate binding site residues on a protein surface by using sequence profile and accessible surface area of spatially neighboring surface residues. 976 non-redundant chains (584 chains from one of the components of homo-dimers, and 196 × 2 chains from both components of hetero-dimers) were trained and tested with 2 fold cross validation. Each of the other component of a homo-dimer was tested by the training set which didn’t contain its homolog. The residues ranked as the top 30% by SVM were further clustered using the clustering method described in [29].

## Authors' contributions

JC and WW developed the concept. JC implemented the codes, did the calculations and drafted the paper. PEB directed the application of the concept. PEB and WW finalized the draft. All authors read and approved the final manuscript.
